# Epidemiological characteristics and spatio-temporal aggregation of severe fever with thrombocytopenia syndrome in Jinan City, China, 2018–2022

**DOI:** 10.1371/journal.pntd.0011807

**Published:** 2023-12-22

**Authors:** Shang Gao, Xingyi Geng, Qingbin Lu, Shanzheng Wu, Zhaoxia Shan, Caiyun Chang

**Affiliations:** 1 Jinan Municipal Center for Disease Control and Prevention, Jinan, China; 2 Center for Infectious Diseases and Policy Research & Global Health and Infectious Diseases Group, Peking University, Beijing, China; 3 Key Laboratory of Epidemiology of Major Diseases (Peking University), Ministry of Education, Beijing, China; 4 Centre for Health Management and Policy Research, School of Public Health, Cheeloo College of Medicine, Shandong University, Jinan, China; Chengde Medical University, CHINA

## Abstract

**Background:**

Severe fever with thrombocytopenia syndrome (SFTS) has become a significant public health issue in Jinan City. However, the analysis of epidemiological characteristics and spatio-temporal clustering of SFTS in Jinan has not been studied yet.

**Methods:**

SFTS data from 2018−2022 in Jinan City were obtained from the China Information System for Disease Control and Prevention. Global spatial autocorrelation and local spatial autocorrelation analyses were performed using ArcGIS 10.2 software, and spatiotemporal hotspot area detection was carried out using SatScan 9.6 software.

**Results:**

Between 2018 and 2022, 680 SFTS cases were reported in Jinan City, resulting in 53 deaths and an average case fatality rate of 7.8%. 99.0% of cases occurred between April and October, 91.9% individuals were over 50 years old, and 87.79% were primarily farmers. A positive spatial correlation of SFTS in Jinan was observed (Moran’s I value between 0.135−0.197, P<0.001), indicating spatial aggregation, primarily in Licheng, Zhangqiu, Laiwu, and Gangcheng districts in southeastern Jinan. Spatiotemporal scanning detected one class I and two class II aggregation areas, with the class I aggregation area (RR = 5.66, LLR = 192.547, P<0.001) locating in southeastern Jinan City, comprising 31 towns/streets, and an aggregation time from 13 May 2020 to 13 October 2022.

**Conclusion:**

Spatial and temporal aggregation of SFTS is evident in Jinan. Based on the spatial and temporal distribution and epidemiological characteristics, prevention and control measures such as public education, monitoring, and training should target key populations in high-incidence epidemic areas.

## Author summary

Severe fever with thrombocytopenia syndrome is a serious natural focal disease caused by SFTS virus. At present, it has become one of the main public health problems in Jinan City. Although some epidemiological surveys have been conducted, there is a lack of relevant spatiotemporal statistical research and the latest information. This study conducts statistical analysis of the latest data in the past 5 years to grasp the spatiotemporal clustering characteristics and change trends of SFTS in Jinan City, analyze the geographical environment and social characteristics of hot areas, then to allocate health resources more reasonably, and provide a basis for precise prevention and control of SFTS.

## Introduction

Severe fever with thrombocytopenia syndrome (SFTS) is a new tick-borne infectious disease. It was identified in 2009 in rural areas of Hubei and Henan provinces in central China [1]. The virus, SFTSV, is mainly transmitted through tick bites [2]. It can also spread via contact with blood, body fluids, or secretions and excretions from infected individuals [3], and infected animal to human is also possible [4,5]. Confirmed cases have been reported in many Asian countries, including China, Korea, Japan, Vietnam, Myanmar, Thailand, and Pakistan [6–11]. Most of these cases are detected in China, with Jinan being a major hotspot for SFTS [12].

SFTS mainly presents with fever, gastrointestinal symptoms, thrombocytopenia, and leukopenia [1]. In some cases, the disease progresses rapidly, leading to multi-organ failure and even death [13]. The number of reported SFTS cases in China has increased annually, with both casefatality rate (CFR) remaining high—an average annual rate of 5.11% from 2011−2021 [14–18]. In 2017, the WHO listed SFTS as a priority infectious disease alongside Ebola and Lassa fever [19].

In recent years, geographic information systems and spatial statistics have been widely applied to describe the distribution characteristics and transmission patterns of diseases, which contributes to the timely surveillance and intervention of diseases [20,21]. In this study, we analyzed the spatio-temporal clustering characteristics of SFTS incidence in Jinan City from 2018 to 2022 at the town/streets level. We also examined the geographical environment and social characteristics of hotspot areas to provide a basis for a more reasonable allocation of health resources and accurate prevention and control of SFTS.

## Materials and methods

### Study area

Jinan locates in the middle of Shandong Province, and lies between 36°01′to 37°32′north latitude and 116°11′to 117°44′east longitude, covering an area of 10,244 square kilometers. Resting on Mount Tai in the south, across the Yellow River in the north, Jinan locates at the junction of low hills in central and southern Shandong and alluvial plain in northwestern Shandong, and the terrain is high in the south and low in the north. It has jurisdiction over 10 districts including Lixia, Shizhong, Huaiyin, Tianqiao, Licheng, Changqing, Zhangqiu, Jiyang, Laiwu and Gangcheng, and two counties of Pingyin and Shanghe.

### Data sources

The data on SFTS cases in Jinan City from January 2018 to December 2022 were obtained from the China Information System for Diseases Control and Prevention (CISDCP). Information included the age, gender, occupation, residential address and date of illness onset of the SFTS cases. The diagnosis standard of the SFTS patient was in line with the China SFTS diagnosis and treatment guide published by Ministry of Health, China in 2010 [22]. The demographical data of Jinan City was downloaded from the Jinan Statistical Yearbook (http://jntj.jinan.gov.cn/).

### Methods

Descriptive statistics such as mean and standard deviation (SD) were used for measurement data conforming to normal distribution. Median and interquartile range (IQR) was employed to describe age. Count data were described by frequency and ratio. The chi-square test was applied to compare the distribution of gender and occupation of SFTS, and the Mann-Whitney U test was used to compare age distribution. ArcGIS 10.2 software was utilized for global spatial autocorrelation, local spatial autocorrelation analysis, and map visualization. SatScan 9.6 software was employed for spatiotemporal hotspot area detection, and a *P*-value <0.05 was considered to be significant.

### Spatial autocorrelation

ArcGIS 10.2 software was used to calculate the global Moran’s I coefficient to detect overall spatial autocorrelation. Moran’s I coefficient values range from -1 to 1:when the value is >0, it indicates a positive spatial correlation. The larger the value, the stronger the spatial aggregation. When the value is <0, it indicates a negative spatial correlation. The closer the value is to -1, the greater the spatial variability. When the value is = 0, it indicates spatial irrelevance. The significance of Moran’s I coefficient should be evaluated according to *Z* and *P* values [23].

### Local spatial autocorrelation

ArcGIS 10.2 software was used to calculate Local Indicators of Spatial Autocorrelation (LISA) and analyze local spatial autocorrelation in four regions: high-high aggregation, high-low aggregation, low-high aggregation, and low-low aggregation."High-high" and "low-low" clusters indicate that the observed values in one region are similar to those in the surrounding regions and are high (low) values."High-low" and "low-high" clusters indicate that the observed values in one region are larger (smaller) than the observed values in the surrounding area.

### Spatiotemporal aggregation distribution

Spatiotemporal aggregation analysis considers both temporal and spatial factors by creating a scan window (cylinder) in geographic space. The bottom surface of the cylinder corresponds to the geographic area under study, the height of the cylinder corresponds to the scan interval, and the radius of the cylinder corresponds to the population at risk of the scan. This method involves randomly selecting a case point or a small area center point (e.g., township point) in the study area as the center of the bottom surface of the scan window. The corresponding geographic area (bottom area of the scan window) and the corresponding time interval (height of the scan window) keep changing until a pre-specified upper limit is reached. The log-likelihood ratio (LLR) is then calculated using the actual and expected values of cases inside and outside the scan window, and a statistical test is performed.

Herein, based on the assumption of discrete Poisson distribution of SFTS incidence in time and space, SatScan 9.6 software was applied with a maximum scan radius of 50% of the total population in the spatial dimension and a time interval of "days." The number of Monte Carlo simulations was set to 999. The window with the largest LLR value was selected as the first class of aggregation, and other windows with statistically significant LLR were considered as the second class of aggregation.

## Results

### Epidemiological characteristics

**Fig 1:**
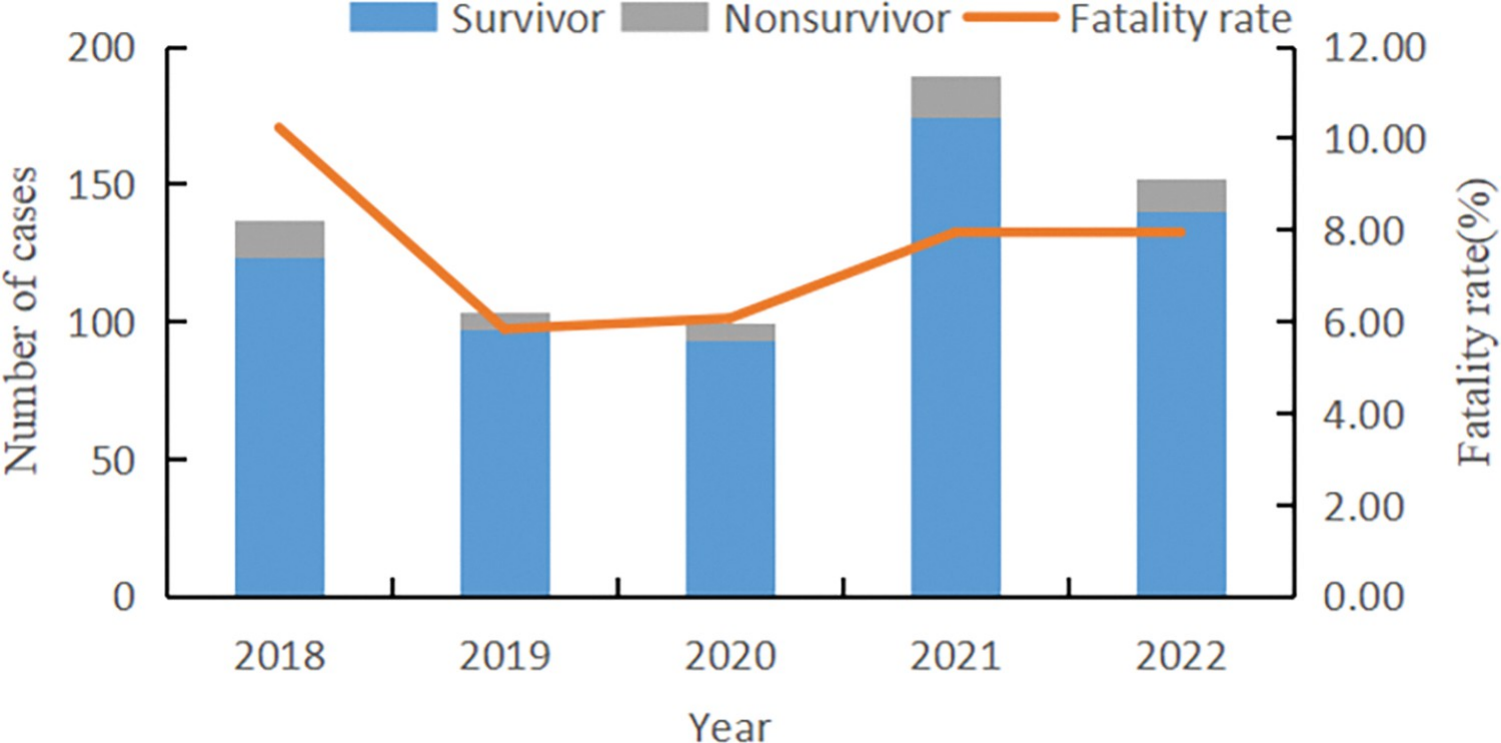
The number of SFTS cases and Casefatalityrate in Jinan from 2018 to 2022.

From 2018 to 2022, a total of 680 SFTS cases were reported in Jinan, with 137, 103, 99, 189, and 152 cases in each respective year. During this period, there were 53 deaths, and the average CFR was 7.79% with 14 cases (10.22%) in 2018, 6 cases (5.83%) in 2019, 6 cases (6.06%) in 2020, 15 cases (7.94%) in 2021,and 12 cases (7.89%) in 2022, respectively (**Fig 1**).

Among the 680 cases, 336 were male and 344 were female (**Table 1**). A statistically significant difference of CFR was observed in the gender (10.71% vs. 4.94%, P = 0.005). The majority of cases were farmers (597 cases, 87.79%), followed by retirees (37 cases, 5.44%) and homemakers and non-working individuals (32 cases, 4.71%). There was no statistical difference in the occupational distribution between surviving and fatal cases (P = 0.424). Most cases (625, 91.91%) were older than 50 years, and the median age was 65 (IQR 57–71) years for surviving cases, and 71 (IQR 66–76) years for deceased cases (P<0.001).

**Table 1:** Socio-demographic characteristics of SFTS in Jinan during 2018–2022.

Characteristics	Survivor	Death	Total
**Gender**			
Male, n(%)	300 (47.85)	36 (67.92)	336 (49.41)
Female, n(%)	327 (52.15)	17 (32.08)	344 (50.59)
Sex Ratio	0.92	2.12	0.98
**Occupation**			
Farmer, n(%)	552 (88.04)	45 (84.90)	597 (87.79)
Retired, n(%)	33 (5.26)	4 (7.55)	37 (5.44)
Housework and unemployment, n (%)	28 (4.47)	4 (7.55)	32 (4.71)
Others, n (%)	14 (2.23)	0 (0.00)	14 (2.06)
**Age, years, median(IQR)**	65 (6–91)	71 (66–76)	66 (58–72)

**Fig 2:**
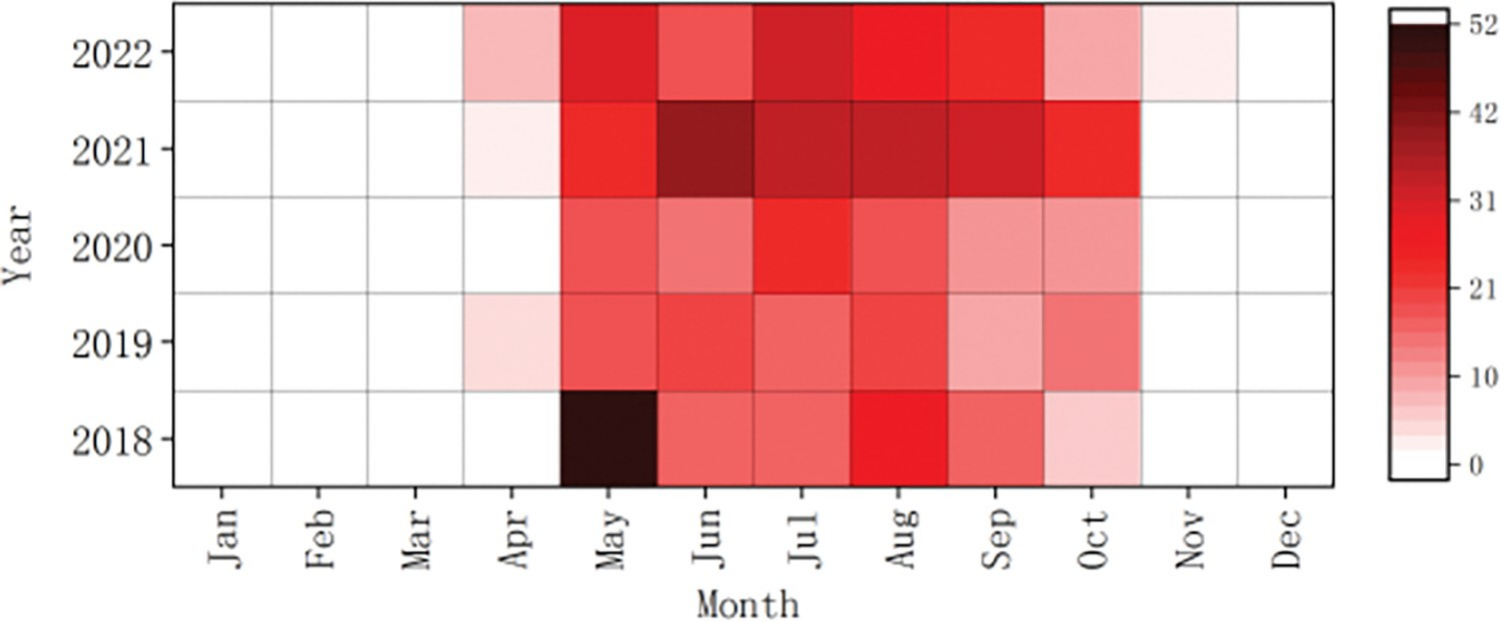
Monthly distribution of SFTS cases in Jinan, 2018–2022.

The heat map revealed that out of the 680 cases, 673 (99.0%) were concentrated in April-October and 498 (73.2%) cases occurred between May and August (**Fig 2**).

**Fig 3:**
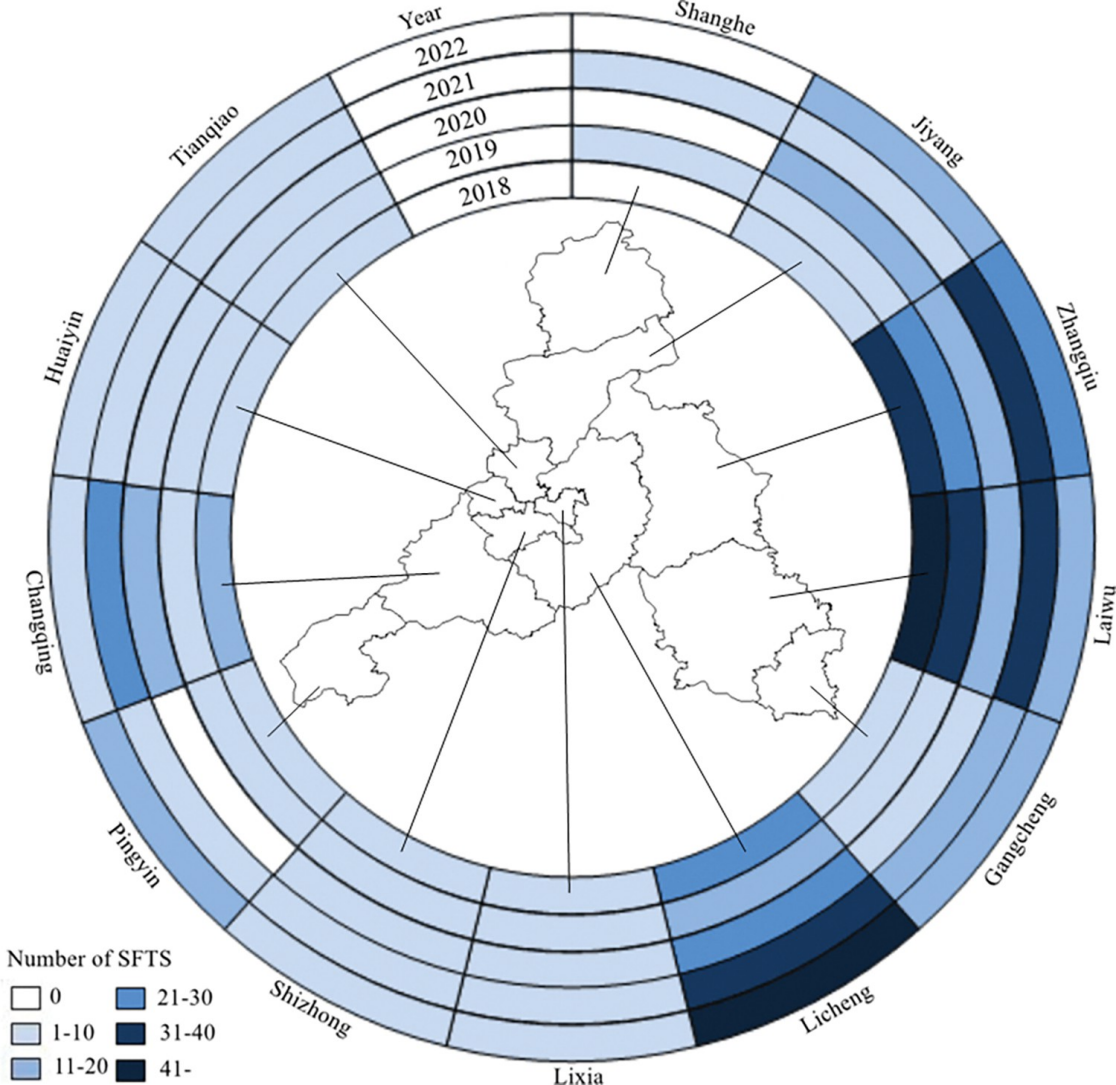
Spatio-temporal distribution of SFTS cases in Jinan, 2018–2022. https://www.sdmap.gov.cn/index.html.

**Fig 4:**
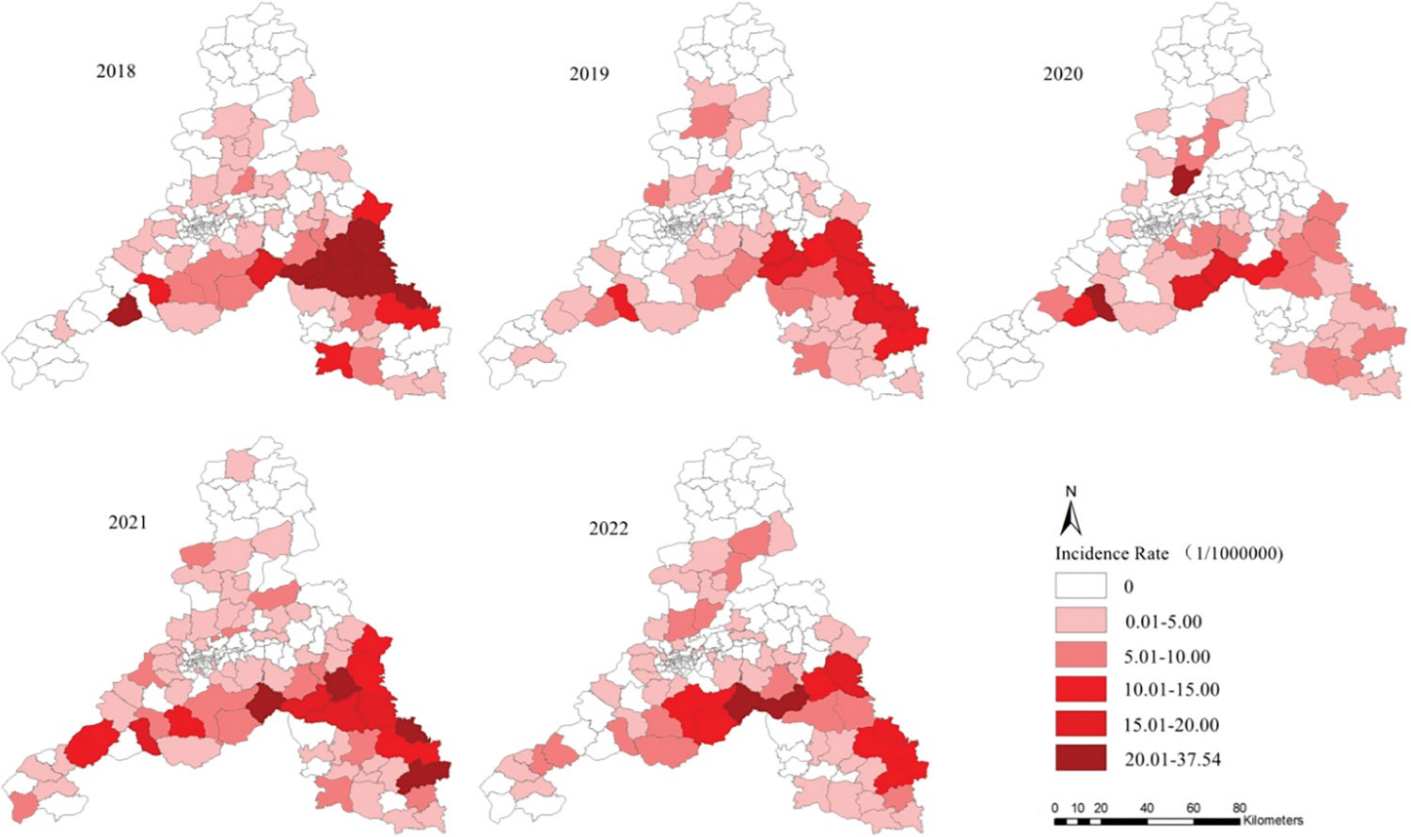
The annual incidence rates of different towns in Jinan, 2018–2022. https://www.sdmap.gov.cn/index.html.

All districts/counties under Jinan City reported SFTS cases druing 2018−2022, except for Shanghe County in 2018, 2020, and 2022, and Pingyin County in 2020. The most cases were reported in LichengDistrict (154), followed by LaiwuDistrict 144 cases, ZhangqiuDistrict 124 cases (**Fig 3**). The top three Areas with incidence rates exceeding 20/100,000 included Guanzhuang Street, WenzuStreet, and Duozhuang Town in Zhangqiu District (**Fig 4**). These areas represented the most severe epidemic locations in Jinan.

### Spatial autocorrelation analysis

The global spatial autocorrelation analysis indicated a positive spatial autocorrelation of the SFTS incidence rate for each year in Jinan City during the study period (Moran’s I index >0 and P<0.05), demonstrating obvious spatial clustering (**Table 2**).

**Table 2:** The Moran’s *I* of global spatial autocorrelation analysis during 2018–2022.

Year	Moran’s*I*	*Z*-Score	*p*-Value
2018	0.161	10.610	<0.001
2019	0.173	11.367	<0.001
2020	0.135	9.065	<0.001
2021	0.197	12.967	<0.001
2022	0.149	10.043	<0.001

### Local spatial autocorrelation analysis

**Fig 5:**
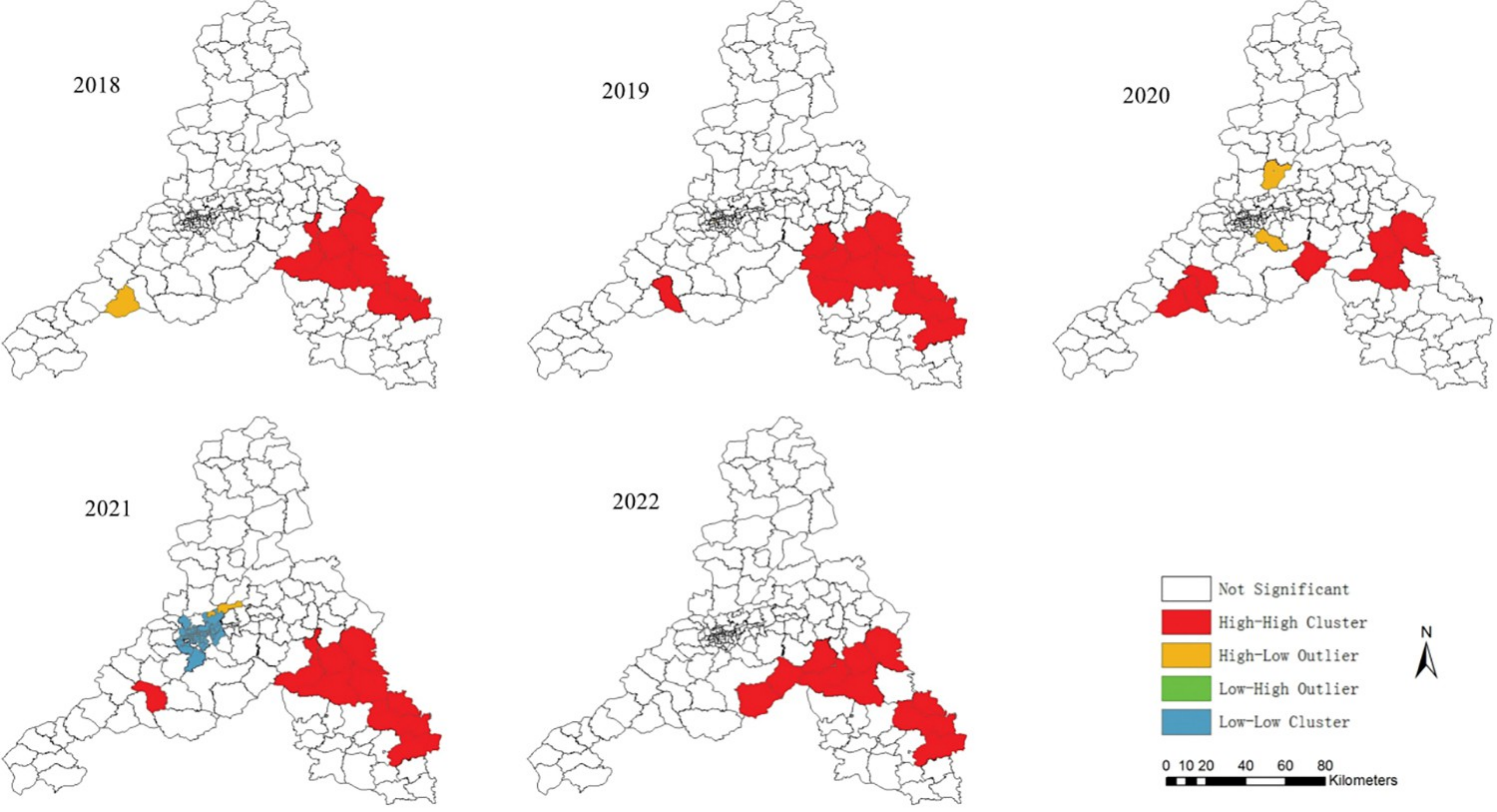
The spatial-temporal of SFTS agglomeration areas in Jinan, 2018–2022. https://www.sdmap.gov.cn/index.html.

The results of local spatial autocorrelation analysis show that there were three types of areas in Jinan City during 2018−2022: high-high aggregation, high-low aggregation and low-low aggregation. High-high aggregation was mainly distributed in LichengDistrict, ZhangqiuDistrict, LaiwuDistrict, GangchengDistrict and ChangqingDistrictin the south of Jinan City. Except for 9 streets in 2018.7 streets in 2020, there are no less than 10 streets in other years. High-low aggregation located in the central and southern parts of Jinan City (**Fig 5**). It was worth noting that some streets/towns in Zhangqiu District and LaiwuDistrict had consistently belonged to high-high clustering areas. Low-low aggregation only occured in the central part of Jinan City in 2021,which may be affected by non intervention measures of COVID-19.

### Space-time cluster analysis

**Fig 6:**
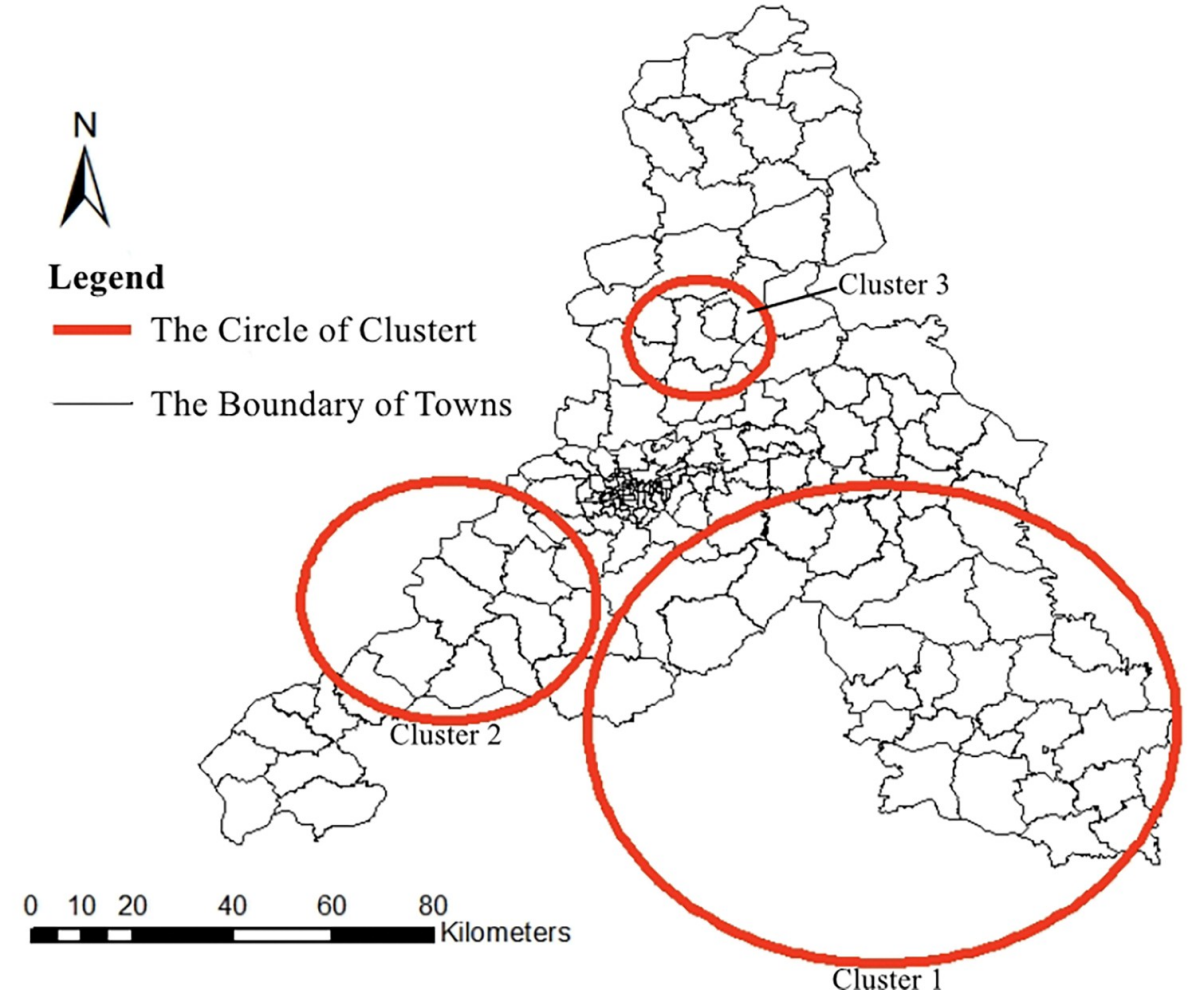
Time-space clusters of SFTS cases at county level in Jinan, 2018–2022. https://www.sdmap.gov.cn/index.html.

As summarized in **Table 3**, the most likely cluster was mainly distributed in the southeastern Jinan City and covered 31 towns/streets (RR = 5.66, LLR = 192.547, P< 0.001), and an aggregation time was from 13 May 2020 to 13 October 2022. The class II aggregation areas 1 covered 12 towns/streets (RR = 6.67, LLR = 26.877, P< 0.001), and an aggregation time was from 29 May 2021 to 17 October 2021. The class II aggregation areas 2 covered 5 towns/streets (RR = 24.43, LLR = 17.850, P = 0.013), and an aggregation time was from 24 September 2020 to 20 October 2020 (**Fig 6**).

**Table 3:** The results of spatial-temporal scanning.

Clusters	Principal	Secondary	
	Cluster 1	Cluster 2	Cluster 3
Coordinates	36.246N,117.447E	36.467N,116.670E	36.938N,117.119E
Radius	47.57km	23.80km	11.58km
Time frame	2020/5/13-2022/10/13	2021/5/29-2021/10/17	2020/9/24-2020/10/20
No. of towns	31	12	5
Number of cases	258	26	8
Expected cases	66.29	4.03	0.33
Annualcases (1/100,000)	5.9	9.7	36.4
Observed / expected	3.89	6.46	24.15
Relative risk	5.66	6.67	24.43
LLR	192.547	26.877	17.850
*P*-value	<0.001	<0.001	0.013

### Tick surveillance

From March to October 2022, Jinan conducted tick surveys for the first time in all counties and districts. The results showed that ticks were detected in all districts and counties except for the northernmost Shanghe County. March to August was the peak period for the occurrence of free ticks in the external environment of Jinan, with an annual average density index of 5.86. In terms of parasitic ticks, a total of 342 animals such as sheep and dogs were monitored, with an average tick index of 7.11 per animal; April is the peak period for host animals to be infected with ticks, with a tick carrying index of 32.86 per animal, followed by May, with a tick carrying index of 15.59 per animal.

## Discussion

In this study, we examined the changes in epidemiological characteristics of SFTS cases in Jinan from 2018 to 2022. Our results revealed that the number of SFTS cases, as well as CFRs, displayed a trend of initially decreasing and then increasing. Several factors might contribute to this trend. Since the discovery of SFTS, health departments have increasingly focused on the disease and implemented various preventive and control measures. These measures included widespread public health education on disease prevention, raising population awareness, and organizing the public to eradicate ticks and improve environmental health. These efforts aim to reduce the density of vector ticks in production and living environments, thereby lowering the incidence of infection [24]. However, the number of reported SFTS cases may also increase as the diagnostic and detection capabilities improve [21].

We observed little difference in the CFR between men and women, but the CFR was higher in men, which may be associated with a more rapid decline in immune function in men compared to women [25]. Age-specific CFRs can further clarify differences among age groups. Generally, these results were the same as previous studies [20,21,26–29]. Epidemiological research indicates that SFTS fatalities are significantly linked to age, gender, underlying health conditions, and other host factors that influence the immune status of an individual. Immune responses to pathogen exposure vary across different ages, with some cytokine and immune cell levels tending to decline as individuals age [30]. This implies that disparities in immune levels between age groups may be associated with increased susceptibility and a higher risk of death among older individuals. The unique distribution of SFTS cases in China is primarily due to social and natural factors [12]. Many young adults in China’s rural areas work away from home throughout the year, leaving the elderly as the primary workforce for rural agricultural activities. The environment of these activities raises the risk of exposure. As a result, the elderly, particularly elderly farmers, are the primary population affected by SFTS.

SFTS is a natural focal disease, with ticks serving as its primary vector. The main transmission mode is through the bite of infected ticks feeding on blood [2,31]. Biological vector monitoring in Jinan revealed that the dominant tick species is the long-horned blood tick, which exhibits a distinct seasonal activity pattern, beginning in spring and lasting through autumn [32,33]. Ticks predominantly inhabit hilly or wooded areas with lush vegetation, and their growth and reproduction are influenced by climatic factors such as vegetation, temperature, humidity, and light. Higher temperatures and humidity levels promote egg-laying by female ticks and the growth and development of juvenile ticks [34]. Seasonal changes in these factors lead to natural fluctuations in tick density [35]. Human outdoor activities, such as farming, mowing, grazing, and traveling, typically occur during summer and autumn. Jinan is situated at the intersection of south-central Lu’s low hills and northwest Lu’s alluvial plains, featuring higher topography in the south and lower topography in the north. Areas with high case numbers, including Zhangqiu, Licheng, Laiwu, Gangcheng, and Changqing districts, locate in the low mountainous hilly regions of southern Jinan. These areas generally have altitudes ranging from 100−400 meters, ample precipitation, and abundant vegetation, making them suitable for the survival and reproduction of Haemaphysalislongicorniscommon in Jinan [20]. As a result, these areas serve as the primary source of SFTS in Jinan.

Moran’s I global autocorrelation analysis results revealed that SFTS cases in Jinan were not randomly distributed, indicating spatial clustering. Further hotspot analysis at the street/town level demonstrated that hotspots extended from the southeast to the west, primarily concentrated in the southern low-hill areas. These regions have abundant vegetation and are suitable for tick survival and reproduction. Human activities such as farming, mowing, grazing, and traveling increase exposure opportunities. Spatio-temporal scan analysis identified three high-risk aggregation areas, which were generally consistent with local spatial autocorrelation results but also exhibited some differences. Both Type I and Type II aggregation area“1”were located in the low mountainous hilly regions of southern Jinan, the same as the local spatial autocorrelation hotspots. However, the Type II aggregation area “2” was situated in Jinan’s central Huangpan plain area, suggesting that besides the low mountainous hilly regions, plain areas can also be sources of SFTS. Ticks can reach other areas through the migration of host animals, transportation, or the natural spread of ticks. Once ticks have spread, controlling new epidemic sites becomes difficult, especially in the absence of more effective prevention and control measures.

This study utilizes surveillance data from the entire Jinan region, and the findings may prove valuable for public health departments in designing and implementing SFTS intervention programs within Jinan. Our study also had some limitations. First, the data came from the national disease surveillance information reporting management system, which may not account for all patients with mild symptoms or undiagnosed SFTS. Second, we could not obtain data on factors like SFTSV carriage in ticks and meteorological factors, which may be associated with SFTS occurrence.

## Conclusion

The SFTS epidemic in Jinan Cityduring2018−2022 displayed a fluctuating trend. Cases primarily occurred between April and October and mainly affected elderly individuals, and farmers. The spatial distribution of SFTS in Jinan City was not random, with high-value aggregation areas mainly in the low mountainous areas in the south. Spatial and temporal scans detected aggregation areas in the central Yellow Pan Plain area in addition to southern low-hill areas. Health education for key populations in high-incidence areas should be emphasized, alongside training disease control and medical personnel, and strengthening tick surveillance for prevention and control work. Prevention measures should be tailored for different populations and areas, considering unique characteristics and risks. Future research should address current study limitations, such as obtaining data on SFTSV carriage in ticks and meteorological factors, to better understand and control the SFTS epidemic.
